# Case report: Ultrasound misdiagnoses atypical parathyroid adenoma as malignant thyroid tumor

**DOI:** 10.3389/fonc.2024.1375373

**Published:** 2024-05-31

**Authors:** Yilin Hou, Lei Zhao, Jiayue Sun, Yun-Fei Zhang

**Affiliations:** ^1^ Department of Ultrasound, The First Hospital of China Medical University, Shenyang, Liaoning, China; ^2^ Department of Ultrasound, The First Affiliated Hospital of China Medical University, Shenyang, Liaoning, China

**Keywords:** atypical parathyroid adenoma, ultrasonic diagnosis, diagnostic errors, thyroid malignancy, case report

## Abstract

Atypical Parathyroid Adenoma (APA) is a type of tumor that lies somewhere between parathyroid adenoma and parathyroid carcinoma. It often affects adults over the age of 60, and the clinical symptoms are consistent with those of hyperparathyroidism. This condition has a low occurrence, and its ultrasonographic signs are strikingly similar to thyroid malignant tumors, making it easily misdiagnosed. As a result, a case of APA ultrasonography misdiagnosis admitted to our hospital was recorded in order to serve as a reference point for APA diagnosis.

## Introduction

APA is a kind of parathyroid adenoma that has a malignant potential between parathyroid adenoma and parathyroid carcinoma (1). It is readily misinterpreted as a malignant tumor because it lacks evident invasion of arteries and capsules, distant metastases, and other symptoms similar to parathyroid carcinoma (2). A instance of APA ultrasonography misdiagnosis of a thyroid malignant tumor in our institution is described here.

**Figure 1:**
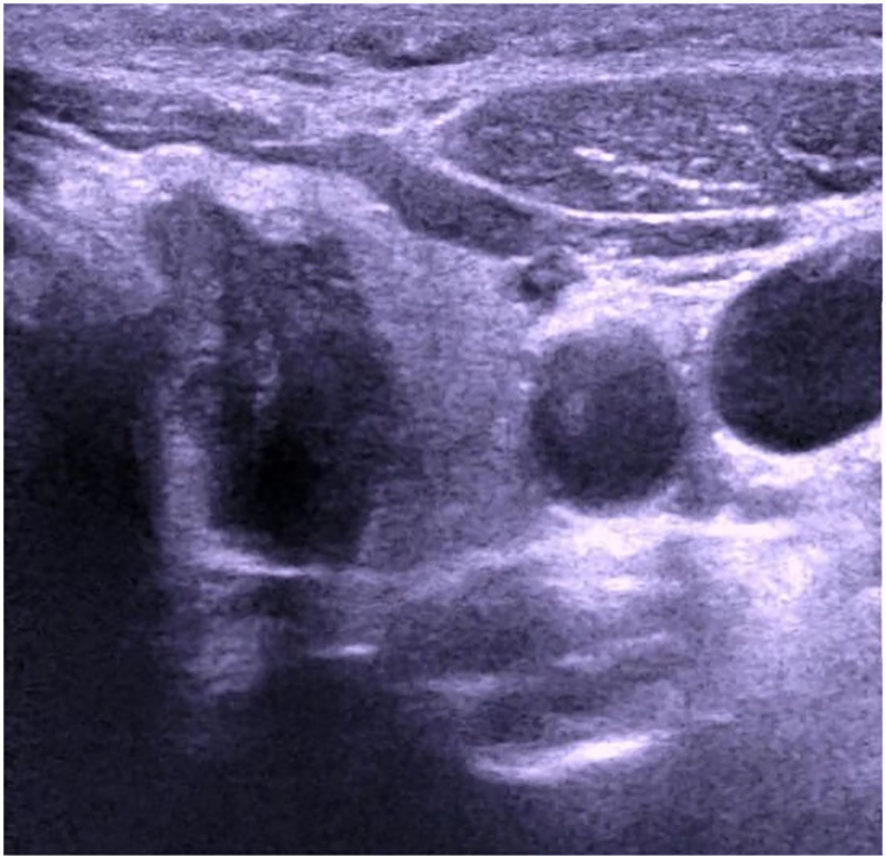
Ultrasound image of atypical parathyroid adenoma misdiagnosed as “thyroid malignant tumor”.

**Figure 2:**
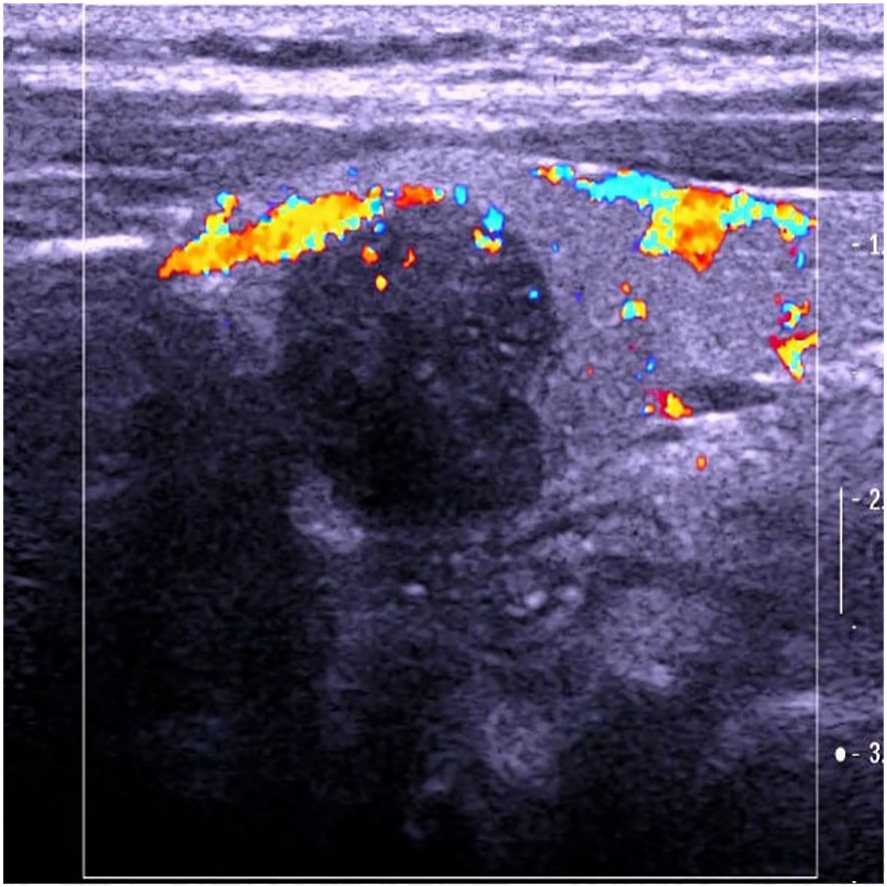
Color Doppler of atypical parathyroid adenoma misdiagnosed as “thyroid malignant tumor”.

**Figure 3:**
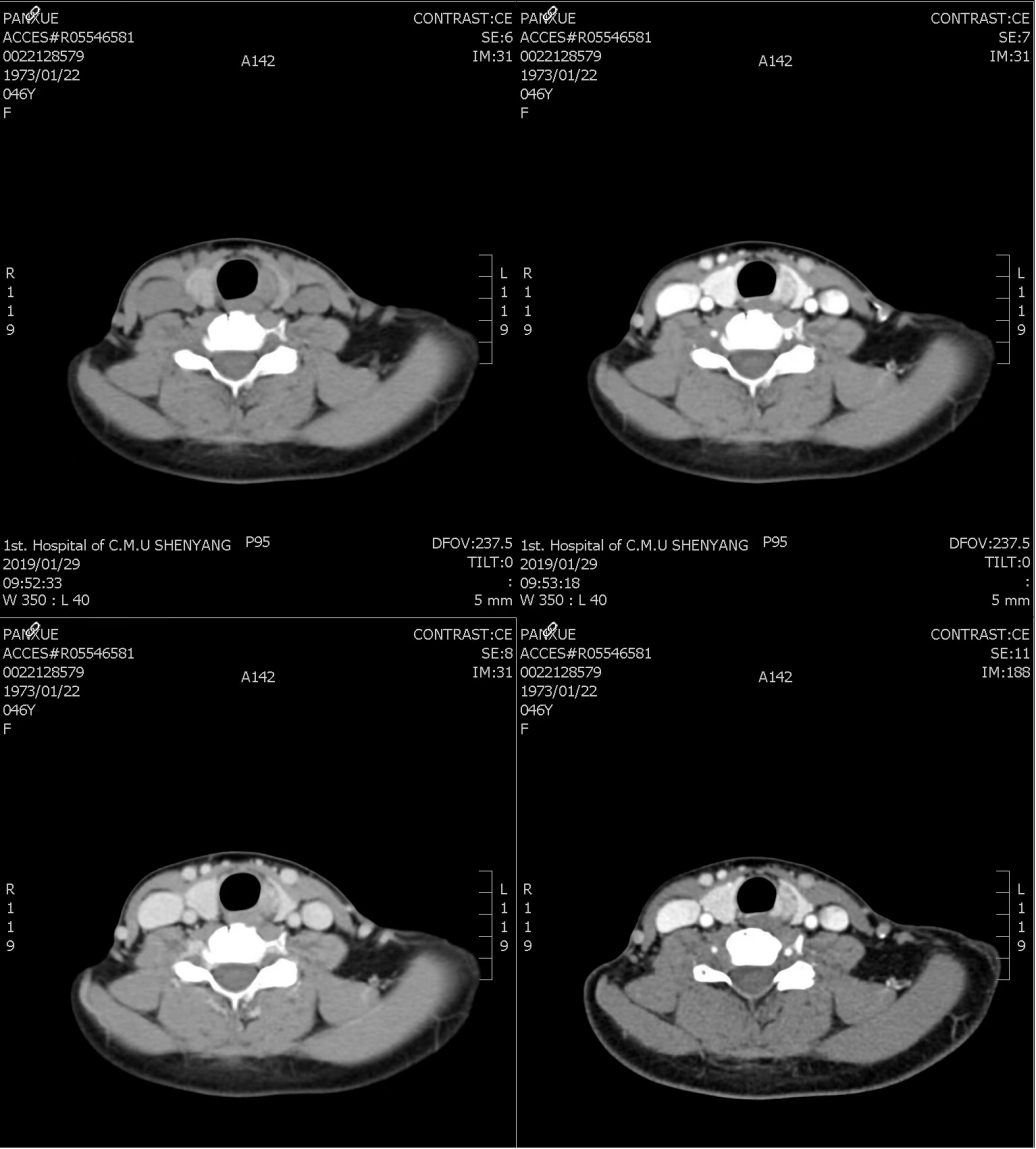
Enhanced CT image of atypical parathyroid adenoma in the neck.

**Figure 4:**
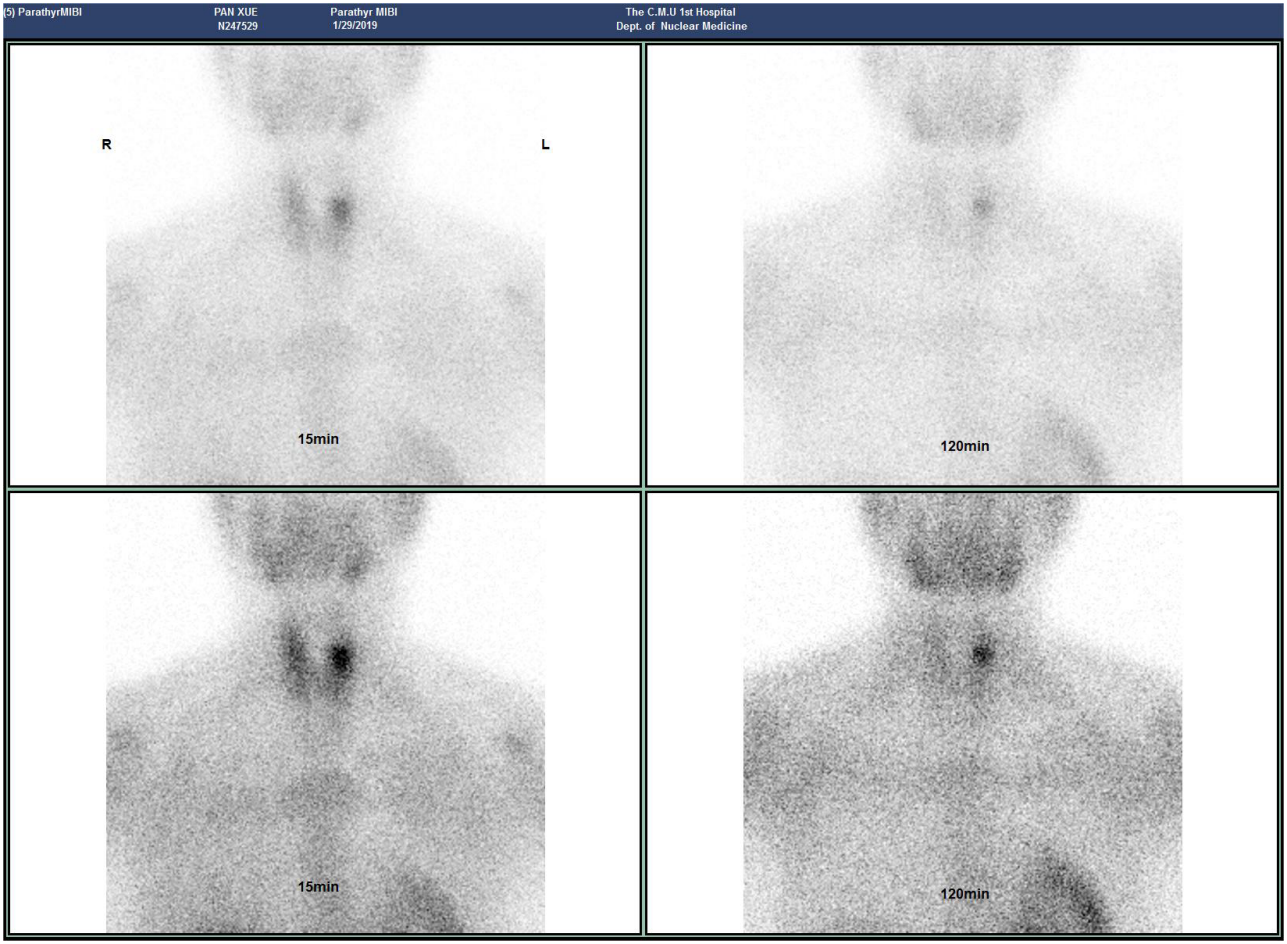
The image of parathyroid ECT scan.

**Figure 5:**
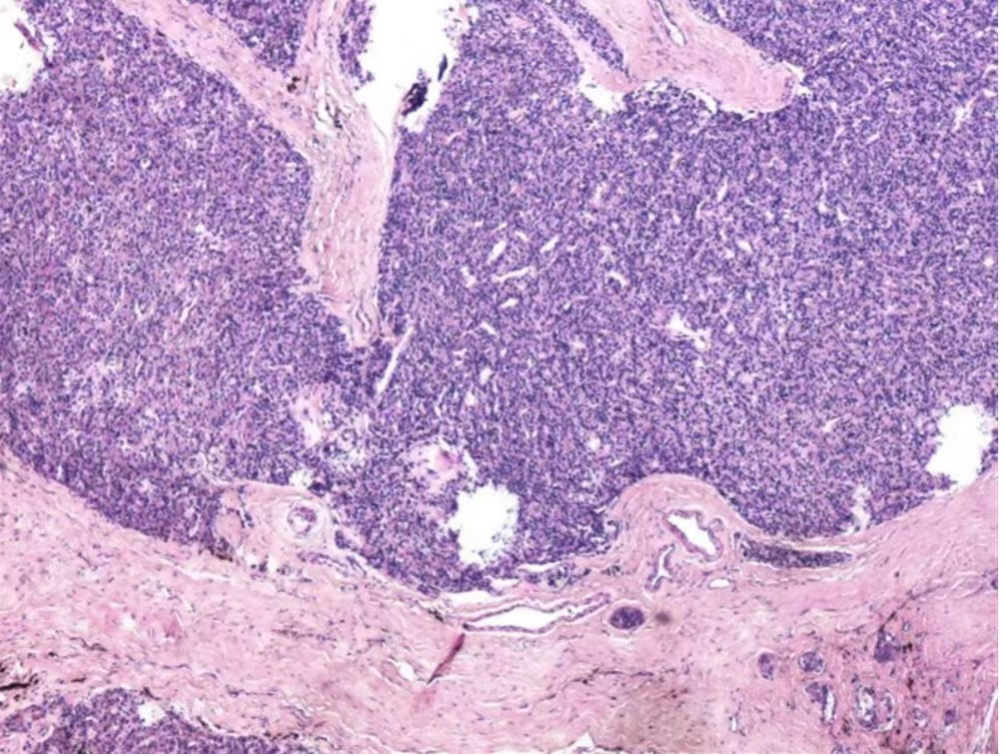
The pathologic images of the surgical specimen.

The patient, a 46-year-old female, reported to the First Affiliated Hospital of China Medical University with the major complaint of “thyroid mass discovered during physical examination for two years.”. Physical examinations: The neck is symmetrical, with the trachea in the center, and a tough lump around 2cm in diameter on the left front of the neck. The nodule’s surface is smooth, no tenderness, and it moves up and down when swallowed. There were no evident swollen lymph nodes on the neck, and no clear vascular murmurs were heard during neck auscultation. Ultrasound results show a 1.79×1.15×1.61cm nodule behind the upper pole of the left lobe of the thyroid gland. The nodule is solid hypoechoic with uneven internal echoes, visible punctate strong echoes with unclear meanings, nodules in a vertical position, irregular edges, lobulated, and clear boundaries. The relationship between the inner side and the trachea is close, and the boundary is unclear (**Figure 1**). A small color blood flow signal can be seen at the margin of the CDFI nodule (**Figure 2**). Lymph nodes on both sides of the neck showed no structural abnormalities on ultrasonography. The ultrasound revealed a left lobe thyroid nodule (ACR-TIRADS 5type) (3).Cervical enhanced CT revealed a left medial paratracheal thyroid space occupying lesion (**Figure 3**). ECT diagnosis: Increased distribution of imaging agents in the upper left lobe of the thyroid, the possibility of hyperparathyroidism is high (**Figure 4**). Preoperative laboratory results showed iPTH: 46.49 pmol/L (reference range: 0.66–12.00), serum calcium: 2.74 mmol/L (reference range: 2.17–2.57), phosphorus: 0.77 mmol/L (reference range: 0.81–1.52), magnesium: 0.88 mmol/L (reference range: 0.78–1.28), creatinine: 47 umol/L (reference range: 45–84), serum albumin: 40. 9 g/L (reference range: 40–55). Biochemical findings indicated that the patient had raised iPTH, elevated blood calcium, and decreased blood phosphorus, which, together with imaging and laboratory tests, consider the possibility that the tumor was from the parathyroid gland and high malignancy probability. Because the patient was nervous and concerned about the risk of tumor cell spillage, she refused to do FNA and wanted to accomplish the treatment through surgery as soon as possible. As a result, the patient was admitted and had surgery under general anesthesia. During the operation, the mass was found in the upper pole of the left lobe of the thyroid gland, with a diameter of about 1.8cm, soft, invading the gland, and being tightly attached to the trachea and recurrent laryngeal nerves. The intraoperative pathology included a parathyroid adenoma with capsule invasion and localized capsule penetration. The thyroid surgeon considered the intraoperative pathological suggestion of capsule invasion, which was inconsistent with the typical parathyroid adenoma and had malignant potential, so the left upper parathyroid mass and the invaded left thyroid lobe were resected, and the anterior laryngeal lymph node dissection. A postoperative pathological examination using light microscopy revealed a tightly packed cluster of tumor cells with deep stained nuclei and copious cytoplasm, as well as an increase in the number and size of localized thyroid follicles and fibrous tissue growth.Paraffin and immunohistochemistry can be used to further detect or exclude thyroid follicular cancer. Immunohistochemical results: CK19 (weak+), Synaptophysin (-), Galectin-3 (+), Tg (-), TTF-1 (-), PTH (++), Ki-67 (5%), Calcitonin (-), ChromograninA (+), CD56 (-). The final pathological diagnosis was an atypical parathyroid adenoma (**Figure 5**). After surgery, the patient was examined again, and the findings showed: iPTH:3.44pmol/L, calcium: 2.05 mmol/L, phosphorus: 0.94 mmol/L, and magnesium: 0.79 mmol/L. The patient received regular follow-ups and no recurrence has been observed so far.

## Discussion

APA is an intermediate condition between adenoma and adenocarcinoma with unknown malignant potential. APA is characterized as parathyroid tumors with capsule and vascular invasion but no capsule penetration, lymph nodes involvement, or distant metastasis (4). The start age of APA is around 60 years old (5, 6), with a male to female ratio of about 1:1 (7). Its clinical symptoms are consistent with those of hyperparathyroidism, such as varied degrees of bone pain, osteoporosis, and even fractures, polyuria, urinary system stones, abdominal pain, bloating, and so on (4). Abnormal laboratory tests include high blood calcium, low blood phosphorus, elevated serum PTH, and elevated AKP levels. The preoperative laboratory evaluation in this patient revealed increased blood calcium, decreased blood phosphorus, and raised iPTH concentrations, which are consistent with the biochemical details of typical APA.

Histopathological features of APA include a broad band of collagen fibers that can bind to and thicken the capsule. Tumors may have a capsule and have a distinct trabecular growth pattern, with projecting nuclei, mitotic activity, and nuclear polymorphism (8), but no metastasis or infiltration of surrounding tissues. According to the pathological findings in this case, the cancer cells are tightly clustered, with strong staining of nuclei and copious cytoplasm. They are accompanied by an increase in the number and size of localized thyroid follicles, as well as fibrous tissue hyperplasia, which is consistent with APA’s histopathological characteristics.

Parathyroid lesions are readily misdiagnosed by ultrasonography because normal parathyroid glands are tiny and cannot make a good reflecting contact with surrounding tissues, making ultrasound difficult to display; Second, the number and location of parathyroid glands vary significantly, and the anatomical relationship of the neck is complex; The third reason is that the echo of the parathyroid gland is close to the thyroid gland and difficult to identify, making it easy to misdiagnosed as thyroid disease or missed diagnosis. Based on this example, the following are the grounds for misdiagnosis: 1. Most parathyroid lesions have a membrane boundary with normal thyroid glands and show as arc-shaped hyperechogenicity. In this case, there is no clear boundary between the nodule and the thyroid gland, and the lesion seems to be within the thyroid gland on ultrasound pictures, leading to a misdiagnosed as a thyroid origin lesion. 2. The nodule is solid, hypoechoic, vertical, lobulated, and has unclear punctate strong echoes (microcalcification cannot be identified), all of which suggest thyroid cancer. According to the ACR-TI-RADS classification standard, it has reached 5 categories, whereas the Chinese TIRADS classification standard (C-TIRADS) has reached 4c categories, so it is misdiagnosed as a malignant tumor; 3. In this case, due to highly suspicious malignant nodules on imaging, Neither preoperative gene analysis was performed, nor adequately considering factors other than imaging, which is another important reason for misdiagnosis.

APA and thyroid tumor intersect in the two-dimensional audiogram, making it difficult to diagnose them solely on the two-dimensional audiogram, which can lead to misdiagnosis and missed diagnoses. Some studies have demonstrated that combining CEUS, elastography, and 99mTc MIBISPECT/CT multimodal imaging diagnostic approaches can greatly increase the accuracy of parathyroid lesions (9). Parathyroid lesions can be seen on contrast-enhanced ultrasound with quick in and out of the contrast agent, as well as significantly uniform or uneven boosting of nodules (10). Combined with this patient’s enhanced cervical CT, a semicircular soft tissue density shadow was noticed beside the left medial thyroid trachea, and the enhanced scan revealed uneven enhancement (**Figure 3**). On elastography, APA is frequently seen as a “stiff edges” and a “colored lesion”. The term “stiff edges” refer to the increased rigidity of the peritumoral tissue when compared to the surrounding soft tissue, and “colored lesion” alludes to the diseased tissue’s uneven and colorful appearance (11). This is closely related to APA’s pathological traits, which include increased connective tissue, adherence to neighboring structures, and band fibrosis. 99mTc-MIBI SPECT/CT fusion imaging can detect both the functional status of the lesion and the interaction between the lesion and neighboring tissues, and a localized radioactive focused focus can be observed in the lesion site (12). Image of the parathyroid glands in this patient: early imaging was seen 15 minutes after medication injection, the imaging agent in the upper left lobe of the thyroid gland was dispersed in the thickening area, while the other billet imaging agent was spread normally. Delayed imaging 2 hours after medication injection resulted in no significant decrease in upper left thyroid lobe thickness, and reduced dispersion of the remaining imaging agent (**Figure 4**). The absence of a multimodal imaging evaluation in this case is also one of the causes of disease misdiagnosis.

High-impact germline CDC73 mutations have been demonstrated to enhance the incidence of parathyroid cancer via altering the C-terminal domain(CTD) of parafibromin (13). As a result, CDC73 mutation screening in patient blood samples is critical when the lesion’s origin is suspected to be parathyroidal. Furthermore, the parathyroid gland is the primary organ involved in multiple endocrine tumor syndrome (MEN) including two main forms, MEN type 1 (MEN1) and type 2 (MEN2). MEN1 is characterized by the combined occurrence of parathyroid, pituitary and pancreatic neuroendocrine tumors, whereas MEN2 features medullary thyroid cancer in association with phaeochromocytoma and parathyroid tumors. Although both MEN1 and MEN2 are autosomal dominant disorders, they have contrasting molecular etiologies: MEN1 results from inactivating germline mutations of the MEN1 tumor suppressor gene on chromosome 11 (14), whereas MEN2 results from activating mutations in the RET proto-oncogene. Fully understanding if patients have hyperparathyroidism or MEN family history aids in the early diagnosis or treatment of APA.

In summary, APA can be difficult to identify from thyroid malignant tumors in ultrasound presentations, and a thorough analysis should be performed using a combination of multimodal imaging diagnostic approaches and laboratory assays. The majority of APAs have a good prognosis and seldom have tumor recurrence or metastasis (15). As a result, early and accurate diagnosis and treatment of APA are critical to patient outcomes.

## Data availability statement

The original contributions presented in the study are included in the article/supplementary material. Further inquiries can be directed to the corresponding author.

## Ethics statement

Written informed consent was obtained from the individual(s) for the publication of any potentially identifiable images or data included in this article.

## Author contributions

YH: Formal analysis, Data curation, Writing – review & editing, Writing – original draft. LZ: Writing – review & editing, Resources, Data curation. JS: Writing – review & editing, Formal analysis, Data curation. YZ: Writing – review & editing.
